# Burden and Risk Factors of Brain Metastases in Melanoma: A Systematic Literature Review

**DOI:** 10.3390/cancers14246108

**Published:** 2022-12-12

**Authors:** Xiang-Lin Tan, Amy Le, Huilin Tang, Madeline Brown, Emilie Scherrer, Jiali Han, Ruixuan Jiang, Scott J. Diede, Irene M. Shui

**Affiliations:** 1 Merck & Co., Inc., Rahway, NJ 07065, USA; 2 Richard M. Fairbanks School of Public Health, Indiana University, Indianapolis, IN 46202, USA; 3 Integrative Precision Health, LLC, Carmel, IN 46032, USA; 4 Seagen Inc., Bothell, WA 98021, USA

**Keywords:** melanoma, brain metastases, risk factors, proportion

## Abstract

**Simple Summary:**

Brain metastases are common with severe consequences in patients with melanoma. We conducted a systematic literature review to evaluate the proportion of melanoma patients diagnosed with brain metastases and to summarize risk factors of melanoma brain metastases. These results may contribute to our understanding of the risk and associated factors of brain metastases in melanoma patients.

**Abstract:**

Melanoma can frequently metastasize to the brain with severe consequences. However, variation of melanoma brain metastases (MBM) development among populations is not well studied, and underlying mechanisms and risk factors for MBM development are not consistently documented. We conducted a systematic literature review (SLR) including a total of 39 articles to evaluate the proportion of melanoma patients who are diagnosed with, or develop, brain metastases, and summarize the risk factors of MBM. The average proportion of MBM was calculated and weighted by the sample size of each study. Meta-analyses were conducted for the selected risk factors using a random-effects model. The proportion of MBM at diagnosis was 33% (975 with MBM out of 2948 patients) among patients with cutaneous melanoma (excluding acral) and 23% (651/2875) among patients with cutaneous mixed with other types of melanoma. The proportion at diagnosis was lower among populations with mucosal (9/96, 9%) or uveal (4/184, 2%) melanoma and among populations outside the United States and Europe. Meta-analysis demonstrated that male vs. female gender and left-sided tumors vs. right-sided were significantly associated with increased risk of melanoma brain metastases. These data may help clinicians to assess an individual patient’s risk of developing melanoma brain metastases.

## 1. Introduction

The incidence of melanoma has been increasing over recent decades, especially among white patients in the United States, Canada, Northern Europe, Australia, and New Zealand [1]. Melanoma is the third most likely cancer to cause brain metastases (behind non-small cell lung cancer and breast cancer) [2]. The risk of MBM in advanced melanoma increases with disease duration. Approximately 20–40% of patients have been reported to have brain metastasis at the time of diagnosis of metastatic melanoma, and more than 50% develop brain metastasis during the course of the disease [2,3,4,5]. In patients with stage IIIB and IIIC melanoma, a retrospective analysis of data from a large multi-institutional trial investigating adjuvant treatment identified that 15% of patients developed MBM as a component of melanoma relapse, which occurred predominantly in the first three years after surgery [3]. A study that included patients with stage III melanoma from two large melanoma databases in the United States and Australia found that, overall, 16.7% of patients were diagnosed with MBM during follow-up, and the incidence of MBM was 3.6% at 1 year, 9.6% at 2 years, and 15.8% at 5 years [6]. Nevertheless, the variation of MBM development among populations is not well studied, and the underlying mechanisms for MBM development are not consistently documented.

Several studies have investigated risk factors of MBM, such as age, race, gender, location of primary tumor, histologic subtype, BRAF mutation status, and tumor stage. The presence of ulceration at the primary melanoma site has been linked to MBM development [4]. A better understanding of the risk factors for developing MBM may be helpful for early detection and treatment of asymptomatic MBM, potentially leading to improved outcomes.

In this systematic literature review (SLR) and meta-analysis, we aimed to summarize the burden of MBM, specifically the proportion of stage IV patients who are diagnosed with MBM and the proportion of Stage II–IV melanoma patients who develop MBM during the course of the disease. Further, the SLR also summarized risk factors for being diagnosed or developing MBM.

## 2. Methods

### 2.1. Study Design and Search Strategy

This study was performed in accordance with the Preferred Reporting Items for Systematic Reviews and Meta-Analysis (PRISMA) guidelines, and was not registered. Relevant studies with full-text articles published in English since 2015 were identified by searching the following databases: EMBASE, MEDLINE, Cochrane Register of Controlled Trials, and Cochrane Review on 1 November 2020. One additional publication was included on 1 July 2022. The eligible population was adult patients >18 years with stage II–IV melanoma and any histology who develop (Stage II–IV) or present with (Stage IV) at least one brain metastasis. Eligible study designs were prospective and retrospective cohort studies, case-control studies, cross-sectional studies, controlled and uncontrolled longitudinal studies (cohorts or case series), with no minimum sample sized required. Eligible outcomes of interest were incidence and prevalence of brain metastasis in melanoma patients and risk factors for MBM.

Two reviewers independently selected studies according to the inclusion criteria and extracted data, with a third independent reviewer available to address any discrepancies and perform a quality check. Bibliographies from review articles were reviewed thoroughly to identify relevant additional studies and trial results.

### 2.2. Data Extraction

Data extracted to summarize the proportion of patients diagnosed or developing MBM included number of patients with MBM and total number of melanoma patients. Data extracted for risk factors of MBM included age, race, gender, BMI, marital status, health insurance, family history of melanoma, location of primary tumor, Breslow depth, presence of ulceration, histologic subtype, BRAF mutation status, stage, biomarkers, presence of extracranial metastases, and history of prior surgical resection. The odds ratios (OR) with 95% confidence intervals (CI) for each risk factor were extracted, noting the comparison and reference groups for each study. Of note, the histologic subtypes extracted included cutaneous, acral, mucosal, and uveal.

### 2.3. Data Analysis

The proportion of MBM at diagnosis was calculated using the number of patients with MBM divided by the total number of stage IV melanoma patients in the study. The proportion was further summarized by study type, melanoma type and geographic location. The proportion of stage II–IV melanoma patients who developed MBM after diagnosis were calculated stratified by stage, study type, melanoma type, and geographic location. If a study reported numbers of stage II–IV melanoma patients that developed MBM after diagnosis separately, the study numbers were included in summary tables. Alternatively, if stage I mixed with other stages or stage II–IV numbers were grouped together, these studies were excluded. The average, weighted by sample size of each study, was calculated for the proportion of MBM.

For the risk factor analysis, OR was obtained directly from the studies or was calculated by the current authors when this information was not provided. The reported adjusted OR was used unless it was not available, in which case the crude OR was used. If the OR was calculated, the ratio of the odds of brain metastases in the risk factor group was divided by the odds of brain metastases in the reference group. If more than one study reported the same factors with the same comparison groups, overall meta-analytic OR for MBM development with corresponding 95% CI were calculated for selected risk factors (gender, laterality (right-sided primary vs. left), Breslow depth (≥2 mm, vs. <2 mm), race (White vs. Non-white), BRAF status (mutation vs. wildtype), and ulceration (presence vs. absence)) using random-effects models to account for varying effect sizes across studies. Meta-analysis was not performed on risk factors that were not well-defined or if there was only one study on those factors. Cochrane’s Q test and the I2 statistic were used to assess heterogeneity between studies, with *p* value < 0.05 for Cochrane’s Q test and I2 > 50% considered cut-offs for significant heterogeneity. The results of the meta-analysis are presented graphically as forest plots with the summary measure and 95% CI noted. Publication bias was assessed by contour-enhanced funnel plots of standard error against the effect estimate. All statistical analyses were performed using STATA (Version 14; Stata Corp., College Station, TX, USA), and statistical significance was defined as the *p* value < 0.05.

## 3. Results

### 3.1. Study Selection

**Figure 1:**
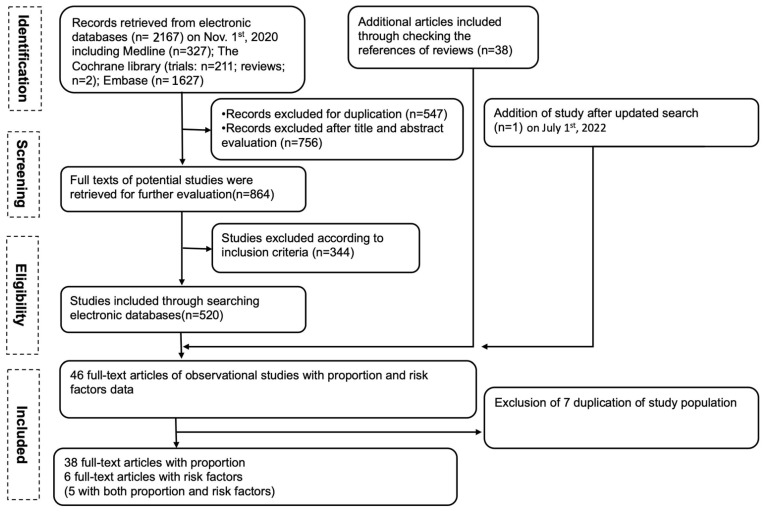
The study flow chart.

Our PRISMA study protocol is presented schematically in Figure 1. Forty-six full-text articles with the outcomes of interest (e.g., proportion and risk factors of MBM) were evaluated, and seven were excluded due to duplication or presenting results on the same population. A total of 39 articles were included in this SLR, and 38 full-text articles were extracted for the proportion results [7,8,9,10,11,12,13,14,15,16,17,18,19,20,21,22,23,24,25,26,27,28,29,30,31,32,33,34,35,36,37,38,39,40,41,42,43,44]. Among of them, 29 studies included patients with MBM at diagnosis (Stage IV) (Supplementary Table S1) and 9 studies reported MBM proportion with brain metastasis occurring after diagnosis (Supplementary Table S2). Six full-text articles were extracted for risk factors (Gardner et al., 2017 only had data available for risk factors of MBM, while the remaining five had data available for both proportion and risk factors) (Supplementary Table S3).

### 3.2. Proportion of MBM

Among the 38 included studies for MBM proportion, 36 were observational studies and two were clinical trials. Five publications (2948 total patients) reported data on cutaneous melanoma separately, with a proportion of MBM at diagnosis of 33% [7,17,31,41,43], and 25% after diagnosis (for Stage IV patients) [26,43]. Fourteen publications included cutaneous mixed with others (with most patients having cutaneous), and the proportion was 23% at diagnosis (*n* = 11 studies, 2875 total patients) [8,10,11,15,16,18,20,22,23,24,29] and 36% after diagnosis for stage IV patients (*n* = 3 studies) [27,39,40] (Table 1). Two studies (184 total patients) included uveal melanoma (MBM proportion at diagnosis of 2%) [31,32], two studies included mucosal melanoma (MBM proportion at diagnosis of 9.4% in 96 total patients) [30,33], and three studies included a mix of acral, mucosal, and others (MBM proportion at diagnosis of 10.5% in 172 total patients) [28,30,42] (Table 1). Overall, the MBM proportion in studies with specific populations of acral, mucosal, and uveal types of melanoma were lower than those observed in studies with cutaneous or mixed type melanoma populations.

When stratified by melanoma type and country/region, the proportion of MBM at diagnosis was found to be highest among those with cutaneous melanoma in the United States (34%) and Europe (35%) (Table 2). In US studies, MBM proportion is relatively higher among patients with cutaneous melanoma (34%, *n* = 2) [7,19], compared to patients with cutaneous melanoma mixed with others (29%, *n* = 2 studies) [11,24]. This pattern was similar in European populations—35% of cutaneous melanoma patients had MBM at diagnosis in two studies [17,31], compared to 17% for mixed (*n* = 5 studies) [8,16,20,23,29]. MBM proportion was 30% at diagnosis in a single study of cutaneous melanoma patients from South America [41], and 29% at diagnosis based on 2 publications on patients with cutaneous melanoma mixed with others from Australia [18,22] (Table 2).

### 3.3. Meta-Analysis for Risk Factors Associated with MBM

Meta-analysis was performed on the following risk factors: gender, laterality (right-sided primary vs. left), and ulceration (presence vs. absence) (Table 3). Retrospective data from a SEER analysis of 2691 metastatic melanoma patients diagnosed in 2010–2013 by Abdel-Rahman et al. showed that male gender compared to female gender was associated with higher odds of MBM at diagnosis (OR 1.34; 95% CI, 1.12–1.60) [7]. Liu et al. also utilized the SEER database, including a total of 62,369 newly-diagnosed melanoma patients of any stage diagnosed between 2010–2014 and found higher risk of MBM at diagnosis among males (OR 1.49; 95% CI, 1.21–1.84) [44]. Given that the study by Liu et al. encompassed a larger population of patients rather than only patients with metastatic disease at diagnosis, we only chose to include this study in the meta-analysis. An analysis by Gardner et al. of 123 cases with MBM from a single institution database and 237 controls matched by initial presenting stage (any stage, but without brain metastases) reported no association with male sex (*p* = 0.12), while our calculated OR is 1.84 (95% CI, 1.15–2.93) [45]. A retrospective cohort study by Maxwell et al. included 225 patients of any stage from a single institution and found no significant association between sex and risk of developing MBM [calculated OR 1.28 (95% CI, 0.69–2.37)] [27]. Our meta-analysis found that male gender was significantly associated with increased risk of MBM (meta-OR, 1.52; 95% CI, 1.26–1.82; *p* < 0.001) (Table 3, Supplementary Figure S1).

Three studies reported the presence of ulceration associated with development of MBM with heterogenous results (Table 3, Supplementary Figure S2). Koelblinger et al. showed that the development of brain metastases was more frequent in patients with ulcerated, than with stage-matched non-ulcerated, primary tumors (pT1-pT4) (17.6 vs. 3.6%, *p* = 0.015), with a calculated OR of 5.87 (95% CI, 1.22–28.16) [37]. Gardner et al. did not find that ulceration was associated with risk of developing MBM (calculated OR, 1.33; 95% CI, 0.83–2.15) [45]. Maxwell et al. found no significant association between ulceration and developing MBM (calculated OR, 0.76; 95% CI, 0.33–1.72) [27]. Our meta-analysis did not show a significant association between ulceration and risk of MBM with an OR of 1.42 (95% CI, 0.64–3.15, *p* = 0.39).

For the association between laterality and the risk of MBM, Abdel-Rahman et al. showed that right-sided localization of primary, compared to left plus unknown/midline laterality, was significantly associated with a lower risk of MBM at diagnosis among patients with metastatic melanoma (OR, 0.73; 95% CI, 0.59–0.91), while no significant association was found by Maxwell et al. for laterality and the risk of developing MBM (OR, 0.63; 95% CI, 0.35–1.25) [7,27]. The overall OR from the meta-analysis was 0.72 (95% CI, 0.59–0.89, *p* = 0.002) (Table 3, Supplementary Figure S3).

### 3.4. Other Risk Factors Associated with MBM Development

Studies examining age (*n* = 2 studies), location of primary tumor (*n* = 2 studies), histology (*n* = 3 studies), and LDH levels (*n* = 2 studies) had heterogenous definitions that could not be combined and summarized by meta-analysis (Table 4). Abel-Rahman et al. and Liu et al. reported on risk of MBM for white vs. non-white ethnicity, however both of these studies utilized the SEER database and thus the population was overlapping. Maxwell et al. demonstrated that patients with BRAF-V600 mutations had a higher risk of developing MBM in multivariate analysis compared to BRAF wild-type for the full cohort (OR 2.24; 95% CI, 1.10–4.58), but a higher risk of developing MBM was not present for the subgroup of patients with BRAF-V600 mutations treated with BRAFi compared to BRAF wild-type, with an OR of MBM of 1.02 (95% CI, 0.40–2.60) [27]. Gardner et al. did not report a difference in BRAF mutation status in patients with MBM (OR 0.95, 0.28–3.2), but no information on BRAFi treatment was available [45]. Therefore, we did not perform meta-analysis for ethnicity and BRAF status (Table 4).

One study by Gardner et al. investigated the association between Breslow depth and the development of MBM (Table 4). This study matched cases with MBM and controls by initial presenting stage (any stage, but without brain metastases) and showed that Breslow depth ≥2 mm compared to <2 mm was significantly associated with higher risk of developing MBM, with an OR of 1.61 (95% CI, 1.02–2.53) [45].

In addition, only a single study assessed the following risk factors: overweight BMI (≥25 vs. normal), unmarried (vs. married), lack of insurance (vs. insured), family history (vs. presence of family history of melanoma), nodal involvement (vs. node positive), T0 stage (vs. other), Stage IV (vs. Stage 0/I/II), no/unknown bone metastases (vs. presence of bone metastases), presence of liver metastases (vs. absence), presence of lung metastases (vs. absence), and no primary surgery or unknown surgery status (vs. primary tumor surgery completed). Therefore, these risk factors were not assessed by meta-analysis, but we summarized the results of these studies in Table 4.

## 4. Discussion

The proportion of MBM was found to be high among patients with melanoma: 33% for cutaneous melanoma at diagnosis and 23% among cutaneous mixed with others at diagnosis. Among patients with stage IV cutaneous melanoma without MBM at diagnosis, 25% of patients later developed MBM. A lower MBM proportion was found among those with non-cutaneous melanoma (mucosal, uveal, or acral), compared to cutaneous melanoma or cutaneous mixed with others.

Prior publications have reported on the associations of patient, tumor, and treatment characteristics with MBM. However, this is the first systematic review and meta-analysis of MBM risk factors. Primary tumor ulceration was previously found to be an independent risk factor for MBM in a large database analysis that included cutaneous melanoma patients of any stage and controlled for stage at diagnosis, primary tumor thickness, and primary tumor anatomic site [46]. Our systematic review identified four studies that investigated the association between ulceration and MBM, however meta-analysis did not show a significant association in terms of risk of developing MBM. This may be related to lack of stratification by stage in the meta-analysis, which was not possible given that some studies stratified by stage while others did not. Additionally, the studies in the meta-analysis included cutaneous melanoma mixed with other histologic types of melanoma (rather than cutaneous melanoma alone).

Additional patient and tumor characteristics were identified as risk factors for MBM in our systematic review, with the meta-analysis confirming the association of gender with increased odds of MBM (male vs. female: OR 1.52, 95% CI = 1.26–1.82). Meta-analysis also found that right-sided tumors were associated with lower odds of MBM (OR 0.72, 95% CI = 0.59–0.89) vs. left-sided or midline tumors, although the causal relationship between these factors and the development of MBM is unclear. There is not any known mechanism explaining the link between gender or laterality of tumor and risk of development of MBM. Further studies are needed to understand the potential biological mechanisms. It is possible that an unknown biomarker or intermediate factor is related to these findings.

The limitations of the present study include the variable inclusion of other types of melanoma, along with cutaneous and heterogenous definitions of risk factors across studies. Given the retrospective nature of the included studies, the associations should be interpreted with caution.

## 5. Conclusions

The proportion of MBM at diagnosis was 33% among patients with cutaneous melanoma (excluding acral), and 23% among patients with cutaneous mixed with other types of melanoma. The proportion at diagnosis was lower among populations with mucosal (9%) or uveal (2%) melanoma and among populations outside the United States and Europe. Meta-analysis demonstrated that male vs. female gender and left-sided tumors vs. right-sided were significantly associated with increased risk of melanoma brain metastases.

Findings from this comprehensive analysis may enhance the knowledge in this field and help clinicians to assess an individual patient’s risk of developing MBM.

## Figures and Tables

**Table 1:** Proportion of MBM by melanoma type and timing of MBM (i.e., at diagnosis (Dx) or after Dx).

Melanoma Type	Timing of MBM	No. of Patients with MBM	No. of Patients	MBM Proportion ^a^	No. of Publications ^b^
Cutaneous	At Dx, Stage IV	975	2948	33%	5 [7,17,31,41,43]
	After Dx, Initial Stage IV	17	68	25%	2 [26,43]
	After Dx, Initial Stage III	66	239	28%	2 [26,43]
	After Dx, Initial Stage II	28	111	25%	1 [26]
Uveal melanoma	At Dx, Stage IV	4	184	2%	2 [31,32]
Mucosal melanoma	At Dx, Stage IV	9	96	9%	2 [30,33]
Acral, mucosal, and others	At Dx, Stage IV	18	172	10%	3 [28,30,42]
	After Dx, Initial Stage IV	7	49	14%	1 [35]
Cutaneous mixed with others ^c^	At Dx, Stage IV	651	2875	23%	11 [8,10,11,15,16,18,20,22,23,24,29]
	After Dx, Initial Stage IV	83	228	36%	3 [27,39,40]
	After Dx, Initial Stage III	14	28	50%	1 [27]

^a^ A weighted average was calculated. ^b^ Out of the 38 studies, two papers reported the proportion of MBM in the cutaneous type and other special type separately. Therefore, we put the cutaneous subgroup into “cutaneous” and the other special type subgroup into “Acral, mucosal, and others” in the table. ^c^ In these studies, the proportion of cutaneous melanoma and other special type was not reported separately, so they are labelled as “cutaneous mixed with others”.

**Table 2:** Proportion of MBM at diagnosis by cutaneous melanoma or cutaneous mixed with others, and geographic location.

Melanoma Type	Location/ Country	No. of Patients with MBM	No. of Patients	MBM Proportion ^a^	No. of Publications ^b^
Cutaneous	United States	946	2797	34%	2 [7,43]
Cutaneous mixed with others ^c^	United States	50	170	29%	2 [11,24]
Cutaneous	Europe	15	43	35%	2 [17,31]
Cutaneous mixed with others ^c^	Europe	125	738	17%	5 [8,16,20,23,29]
Cutaneous	South America	14	46	30%	1 [41]
Cutaneous mixed with others ^c^	Australia	276	955	29%	2 [18,22]

^a^ A weighted average was calculated. ^b^ Studies with missing or mixed geographic location are excluded from this table. ^c^ In these studies, the proportion of cutaneous melanoma and other special type was not reported separately, so they are labelled as “cutaneous mixed with others”.

**Table 3:** Meta-analysis for the selected risk factors associated with MBM development.

Risk Factors	Studies (First Author, Year)	Comparison Groups	OR (95% CI)	Meta-OR (95% CI)
Gender	Liu 2019 [44]	Male vs. Female	1.49 (1.21–1.84)	1.52 (1.26–1.82)
	Gardner 2017 [45]		1.84 (1.15–2.93) ^a^	
	Maxwell 2017 [27]		1.28 (0.69–2.37) ^a^	
Ulceration	Koelblinger 2019 [37]	Presence vs. Absence	5.87 (1.22–28.16) ^a^	1.56 (0.92–2.66)
	Gardner 2017 [45]		1.33 (0.83–2.15) ^a^	
	Maxwell 2017 [27]		0.76 (0.33–1.72) ^a^	
Laterality	Abdel-Rahman 2018 [7]	Right vs. Left + Unknown/Midline	0.73 (0.59–0.91) ^a^	0.72 (0.59–0.89)
	Maxwell 2017 [27]		0.66 (0.35–1.25) ^a^	

^a^ OR was calculated rather than provided by the authors.

**Table 4:** Summary of other risk factors associated with MBM development.

Risk Factors	Studies (First Author, Year)	Comparison Groups	OR (95% CI)
Age	Abdel-Rahman 2018 [7]	<70 vs. ≥70 years	1.47 (1.25–1.73) ^a^
	Gardner 2017 [45]	≥ 60 vs. <60	0.72 (0.45–1.16) ^a^
Location of Primary Tumor	Gardner 2017 [45]	Head and neck vs. Trunk	1.03 (0.59–1.79) ^a^
		Arm vs. Leg	1.89 (0.88–4.05) ^a^
	Maxwell 2017 [27]	Head and neck vs. Other locations	0.48 (0.22–1.02) ^a^
		Trunk vs. Other locations	1.33 (0.70–2.53) ^a^
Histology	Abdel-Rahman 2018 [7]	Melanoma, not otherwise specified vs. Other subtypes	1.93 (1.54–2.41) ^a^
	Gardner 2017 [45]	Nodular vs. Other types	1.98 (1.14–3.44) ^a^
	Maxwell 2017 [27]	Nodular vs. Other types/unknown	0.40 (0.17–0.95) ^a^
Serum LDH levels	Abdel-Rahman 2018 [7]	>10 folds increase vs. <10 folds increase/normal	1.22 (0.60–2.47) ^a^
	Maxwell 2017 [27]	Elevated vs. Not elevated/unknown	0.66 (0.28–1.54) ^a^
Race	Liu 2019 [44]	White vs. non-white (All stages)	3.50 (2.27–5.40)
	Abdel-Rahman 2018 [7]	White vs. non-white (Stage IV)	2.05 (1.23–3.41)
BRAF Status	Maxwell 2017 [27]	BRAF-V600 mutation vs. Wild-type	2.24 (1.10–4.58)
	Gardner 2017 [45]	BRAF mutation vs. Wild-type	0.95 (0.28–3.2) ^a^
Breslow depth	Gardner 2017 [45]	≥2 mm vs. <2 mm	1.61 (1.02–2.53) ^a^
BMI	Richtig 2018 [39]	Overweight vs. Normal weight	0.19 (0.05–0.75) ^a^
Marital Status	Liu 2019 [44]	Not married vs. Married	1.51 (1.24–1.84)
Health Insurance	Liu 2019 [44]	Uninsured vs. Insured	1.93 (1.27–2.89)
T stage	Abdel-Rahman 2018 [7]	T0 vs. Other	1.67 (1.42–1.97) ^a^
N stage	Abdel-Rahman 2018 [7]	N0 vs. N positive/unknown	1.19 (1.01–1.39) ^a^
AJCC Stage	Maxwell 2017 [27]	Stage IV vs. Stage 0/I/II	3.74 (1.45–9.65)
Family History of Melanoma	Gardner 2017 [45]	Yes vs. No	0.57 (0.28–1.13) ^a^
Bone Metastases	Liu 2019 [44]	No/unknown vs. Yes	5.91 (2.12–16.93)
Liver Metastases	Liu 2019 [44]	No vs. Yes	0.60 (0.44–0.81)
Lung Metastases	Liu 2019 [44]	No vs. Yes	0.12 (0.09–0.15)
Surgery to Primary Site	Liu 2019 [44]	Yes vs. No/Unknown	0.08 (0.06–0.10)
Regional Lymph Node Involvement	Maxwell 2017 [27]	Positive vs. Negative/unknown	0.40 (0.20–0.78) ^a^

^a^ OR was calculated rather than provided by the authors.

## Data Availability

The datasets generated during and/or analyzed during the current study are available from the corresponding author on reasonable request.

## Supplementary Materials

The following supporting information can be downloaded at: https://www.mdpi.com/article/10.3390/cancers14246108/s1, Figure S1: Meta-analysis of Association Between Gender (Male vs. Female) and Risk of MBM, Figure S2: Meta-analysis of Association Between Ulceration (Presence vs. Absence) and Risk of MBM, Figure S3: Meta-analysis of Association Between Laterality (Right vs. Left) and Risk of MBM; Table S1: Studies reported MBM proportion with brain metastasis occurred at diagnosis, Table S2: Studies reported MBM proportion with brain metastasis occurred after diagnosis, Table S3: Studies reported risk factors associated with MBM development.
