# Supplementary material for: Burden and Risk Factors of Brain Metastases in Melanoma: A Systematic Literature Review
Source: Cancers (Basel). 2022 Dec 12;14(24):6108. doi: 10.3390/cancers14246108 (PMC9777047; doi:10.3390/cancers14246108)
Supplement: Supplementary file 1 [file cancers-14-06108-s001.zip › cancers-2072934-supplementary.pdf]

Supplementary Table S1. Studies reported MBM proportion with brain metastasis occurred at diagnosis (N = 29, 8 excluded from summary Tables 1-2 are italicized)

Author, Year	Location/ Country	Study Design	Data Source	Melanoma Type and Stage	No. of Patients (Stage IV only)	No. of Patients with MBM	Proportion of MBM
Studies with cutaneous type or majority cutaneous type mixed with other types of melanoma							
Abdel-Rahman, 2018 [7]	United States	Prospective	SEER (2010-distant metastatic)	Cutaneous, Stage IV	2691	906	33.7%
Ahmad, 2015 [8]	United Kingdom	Retrospective	EAP: expanded access programmer	Mixed (82% cutaneous, 8% uveal, 2% mucosal, 3% acral, 5% unknown), 191 patients (99%) had Stage IV, 2 patients (1%) had unresectable Stage III/IV	191	35	18.3%
Arance, 2016 [9]	*Spain*	*Unknown*	*Single center*	*Mixed, Unresectable stage IIIC or stage IV*	*301*	*66*	*NA, stage IIIC and IV not separated*
Atkinson, 2020 [10]	Australia, Netherlands, Spain, Italy, Czech Republic, Lithuania	Retrospective	Named Patient Program (NPP); DESCRIBE II	Mixed, Unresectable Stage III (7.4%) or Stage IV (92.6%)	251	99	39%
Cowey, 2018 [11]	United States	Retrospective, observational	The US Oncology Network	Mixed, Advanced (134 Stage IV, 34 non-metastatic)	134	41	31.0%
Cowey, 2019 [12]	*United States*	*Retrospective, observational*	*US Oncology Network, iKnowMed*	*Mixed, Advanced*	*484*	*155*	*NA, Earlier stages not separated from Stage IV*
Cybulska-Stopa, 2019 [13]	*Poland*	*Retrospective*	*Multicentre*	*Mixed, Skin, mucosal Unresectable or Stage IV*	*318*	*45*	*NA, Earlier stages not separated*

							from Stage IV
Cybulska-Stopa, 2020 [14]	*Poland*	*Retrospective*	*Unknown*	*Mixed, Unresectable (Stage III) or Stage IV*	*287*	*64*	*NA, Earlier stages not separated from Stage IV*
Ferguson, 2018 [15]	Global	Retrospective	Global	Mixed, Metastatic	761	101	13.3%
Guida, 2018 [16]	Italy	Phase II trial	Gruppo Oncologico Italia Meridionale	Mixed [Skin (58), Uveal (3), Mucosal (2), Unknown (6)], Stage IV	69	10	14.5%
Helgadottir, 2019 [17]	Sweden	Familial follow-up	Unknown	Cutaneous, Stage IV	19	5	26.3%
Kim, 2018 [18]	Australia	Cohort	Pharmaceutical Benefits Scheme listing	Mixed, Stage IV	910	262	28.8%
Liu, 2019 [19]	*United States*	*Retrospective*	*Flatiron Health cloud-based longitudinal database*	*Cutaneous, Advanced (Stage III or IV)*	*532*	*96*	*NA, Earlier stages not separated from Stage IV*
Mangana, 2017 [20]	Switzerland	Retrospective cohort	Multicenter	Mixed [SSM (47), NMM (80), ALM (16), LNM (4), desmoplastic (3), amelanotic (7), mucosal (8), other (11), uveal (1), unknown (88)] Stage IV	395	61	15.4%
Moţăţăianu, 2019 [21]	*Romania*	*Cohort*	*1st Clinic of Neurology, Emergency County Hospital of Targu Mures, Romania*	*Mixed, Stage IV (3), or Non-metastatic – specific staging information not provided (18)*	*21*	*8*	*NA, Earlier stages not separated from Stage IV*
Parakh, 2019 [22]	Australia	Retrospective	Unknown	Mixed, Stage IV	45	14	31.1%
Rovere, 2016 [41]	Brazil	Cross-sectional	Single center	Cutaneous, Stage IV	46	14	30.4%

		retrospective					
Funck-Brentano, 2020 [23]	France	Cohort	Single center	Mixed[SSM (6), nodular (5), lentigo maligna (2), acral (1), desmoplastic (2), regressive (2), indeterminate (3), unknown (2)], Stage IV	26	4	15.4%
Wang, 2017 [24]	United States	Cohort	Multicenter (Dana Farber Cancer Institute, Moffitt Cancer Center, Vanderbilt Medical Center)	Mixed [Cutaneous (33), acral (2), mucosal (1), unknown (1), Stage IV	36	9	25.0%
Gorka, 2016 [25]	*Hungary*	*Retrospective*	*Single Center*	*Mixed, Non-metastatic at diagnosis*	*2972*	*333*	*NA, tumor stages not specified*
					225	65	28.9%
Omodaka, 2018 [28]	Japan	Retrospective	Shinshu University Hospital	Mixed, Stage IV	12	3	25.0%
Sandhu, 2021 [43]	United States	Retrospective	Yale-New Haven Hospital tumor registry	Cutaneous vs. Mixed not described, presume cutaneous	106 (Stage IV at Dx)	40 (Stage IV at Dx)	37.7%
Zaragoza, 2016 [29]	France	Cohort	Unknown	Mixed, unresectable Stage III (1) or Stage IV (57)	57	15	26.3%
Zhang, 2019[a] [30]	China	Retrospective	Affiliated Cancer Hospital of ZhengZhou University	Cutaneous, Advanced - Stage III (40) or Stage IV (80)	80	10	12.5%
Kirchberger, 2018[a] [31]	Germany	Retrospective	University Erlangen	Cutaneous, Stage IV	24	10	41.7%
Studies on other types of melanoma							

Jochems, 2019 [32]	Netherlands	Cohort	Dutch Melanoma Treatment Registry	Uveal, Stage IV	175	3	1.7%
Shoushtari, 2017 [33]	United States	Retrospective cohort	Single institution	Mucosal, Unresectable and locally advanced (13) or Stage IV (68)	68	5	7.4%
Chae, 2020 [34]	*Korea*	*Unknown*	*Unknown*	*Oral mucosal, Stage IV (35) or Advanced (39)*	*74*	*7*	*NA, Earlier stages not separated from Stage IV*
Wu, 2020 [42]	Taiwan	Retrospective	Linkou Chang Gung Memorial Hospital	Mixed [Acral (27), cutaneous (14), mucosal (20), others (10), unknown (9)], Stage III (7) or Stage IV (73)	80	5	6.3%
Kirchberger, 2018[a] [31]	Germany	Retrospective	University Erlangen	Uveal, Stage IV	9	1	11.1%
Zhang, 2019[a] [30]	China	Retrospective	Affiliated Cancer Hospital of ZhengZhou University	Mucosal, Stage III (13) or Stage IV (28)	28	4	14.3%

[a]Kirchberger 2018 reported the proportion in the cutaneous type and uveal type separately. Zhang 2019 reported the proportion in the cutaneous type and mucosal type separately.

Supplementary Table S2. Studies reported MBM proportion with brain metastasis occurred after diagnosis (N = 9, 3 excluded from summary Tables 1-2 are italicized)

Author, Year	Location/ Country	Study Design	Data Source	Melanoma Type and Stage	No. of Patients	No. of Patients with MBM	Proportion of MBM
Chang, 2016 [36]	*United States*	*Prospective*	*NYU Interdisciplinary Melanoma Cooperative Group Clinicopathological Biorepository*	*Mixed, Unresectable Stage IIIC or Stage IV*	*31*	*12*	*NA, stage III and IV not separated*
Hanniford, 2015 [26]	United States	Retrospective cohort	Single center (Interdisciplinary Melanoma Cooperative Group database of NYU Langone Medical Center)	Cutaneous, Stages 1-IV followed over time for development of brain metastases – Stage I (74) - Excluded, Stage II (111), Stage III (69), Stage IV (2)	2 (Stage IV); 69 (Stage III); 111 (Stage II)	2 (Stage IV); 27 (Stage III); 28 (Stage II)	100% (Stage IV); 39.1% (Stage III); 25.2% (Stage II)
Koelblinger, 2019 [37]	*Europe (University Hospital of Zurich, Switzerland and Medical University, Salzburg, Austria*	*Unknown*	*Multicenter*	*Mixed - SSM (45), Nodular (48), Acrolentiginous (5), other (14) Non-metastatic (Stage pT1-pT4)*	*56*	*10*	*NA, tumor stages were not specified, only pathologic stage (pT1-pT4) reported*
Larkin, 2019 [38]	*Europe, North America, Latin America, Australia, South Africa, India, South Korea*	*trial, ClinicalTrials.gov (NCT01307397).*	*Multicenter*	*Mixed, Unresectable stage IIIC (2%) or Stage IV (98%)*	*3219*	*753*	*NA, stage IIIC and IV not separated*

Maxwell, 2017 [27]	United States	Retrospective cohort	Single center (Johns Hopkins Hospital Cancer Registry)	Mixed [Nodular (44), SSM (34), Acral/mucosal lentiginous (5), Other (14), Unknown (128),Stage 0-II (62)- Excluded, Stage III (72), IV (36), Unknown (55) and MBM numbers at any time during clinical course]	36 (Stage IV); 28 (Stage III)	16 (Stage IV); 14 (Stage III)	44.4% (Stage IV); 50% (Stage III)
Nakamura, 2020 [35]	Japan	Retrospective	Single institution, University of Tsukuba	Mixed [Acral (20), Superficial spreading melanoma (21), mucosal melanoma (20)], Unresectable stage II (4), Unresectable stage III (9), Adjuvant Stage III (2), or Stage IV (49) – MBM numbers developed during immunotherapy	49 (Stage IV only)	7	14.3%
Richtig, 2018 [39]	Austria	Retrospective, observational	Multicenter	Mixed, Stage IV	76	16	21.1%
Sandhu, 2021 [43]	United States	Retrospective	Single Center (Yale-New Haven Hospital tumor registry)	Cutaneous vs. Mixed not described, presume cutaneous	66 (Stage IV); 170 (Stage III)	15 (Stage IV); 39 (Stage III)	22.7% (Stage IV); 22.9% (Stage III)
Valpione, 2018 [40]	Europe, US, Australia	Retrospective	Multicenter	Mixed, Metastatic	116	51	44.0%

Supplementary Table S3. Studies reported risk factors associated with MBM development

Author, Year	Location/ Country	Study Design	Data Source	Melanoma Type and Stage (No. of patients)	Studied Risk Factors
Abdel-Rahman 2018 [7]	United States	Prospective	SEER (2010-2013)	Cutaneous, Stage IV (N = 2691)	Gender, laterality, age, histology, T-stage, N-stage, serum LDH, race
Gardner 2017 [45]	United States	Case-control	Melanoma Clinical Cancer Database	Cutaneous, Stage IV (N = 360)	Gender, Breslow depth, ulceration, age, primary tumor location, histology, family history of melanoma, BRAF status
Koelblinger 2019 [37]	Europe (University Hospital of Zurich, Switzerland and Medical University, Salzburg, Austria	Retrospective	Multicente r	Mixed - SSM (45), Nodular (48), Acrolentiginous (5), other (14) Non-metastatic (Stage pT1-pT4) (N = 56)	Ulceration
Liu 2019 [44]	United States	Retrospective	SEER (2010-2014)	Cutaneous, All Stages (N = 62369)	Gender, marital status, health insurance, bone metastases, liver metastases, lung metastases, surgery to primary site, race
Maxwell 2017 [27]	United States	Retrospective cohort	Single center (Johns Hopkins Hospital Cancer Registry)	Mixed [Nodular (N= 44), SSM (N= 34), Acral/mucosal lentiginous (N= 5), Other (N= 14), Unknown (N= 128), Stage 0-II (N= 62), Stage III (N= 72), IV (N= 36), Unknown (N= 55) and MBM numbers at any time during clinical course]	Gender, ulceration, laterality, primary tumor location, histology, AJCC stage, regional lymph node involvement, BRAF status
Richtig 2018 [39]	Austria	Retrospective , ob servational	Multicente r	Mixed, Stage IV (N= 76)	BMI

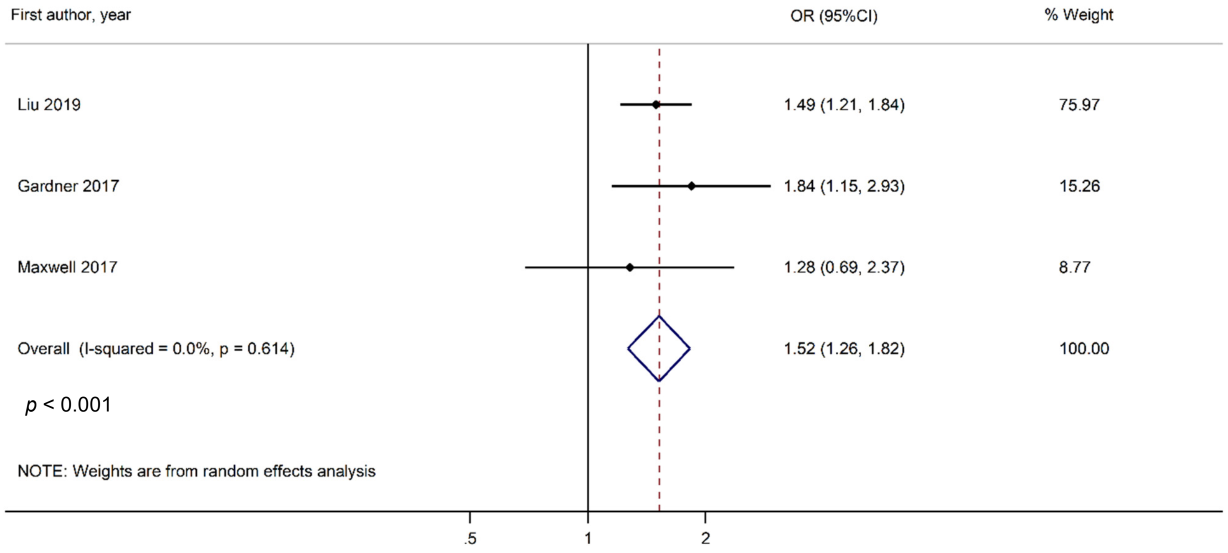

Supplementary Figure S1. Meta-analysis of Association Between Gender (Male vs. Female) and Risk of MBM

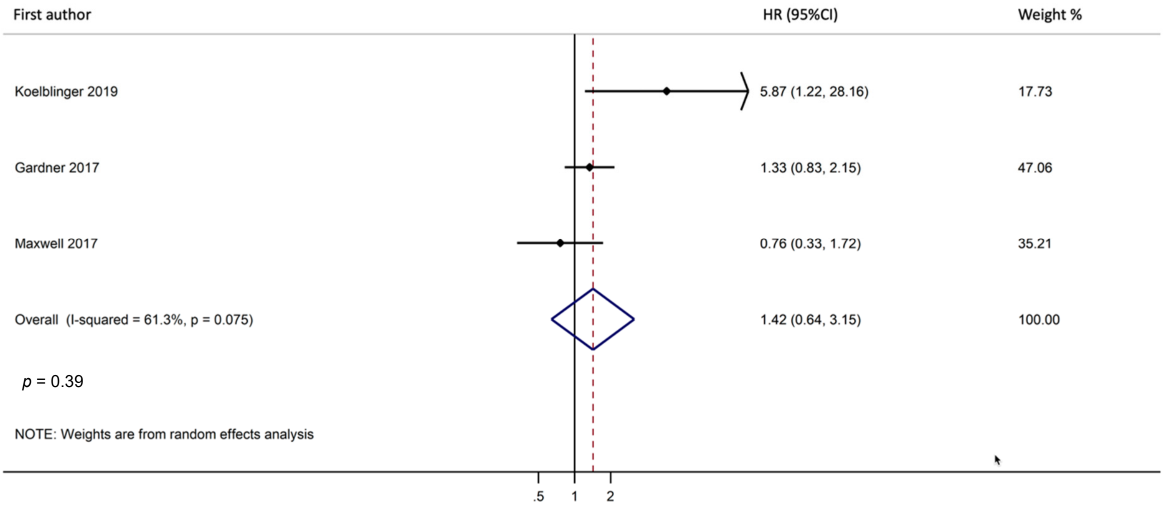

Supplementary Figure S2. Meta-analysis of Association Between Ulceration (Presence vs. Absence) and Risk of MB

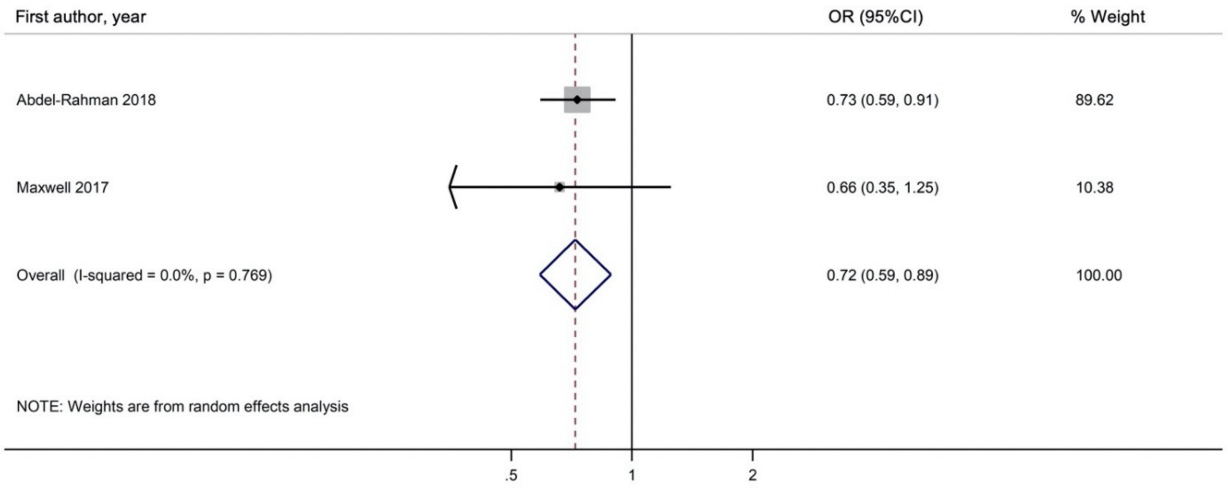

Supplementary Figure S3. Meta-analysis of Association Between Laterality (Right vs. Left) and Risk of MBM
